# Inter-Method Discrepancies in Brain Volume Estimation May Drive Inconsistent Findings in Autism

**DOI:** 10.3389/fnins.2016.00439

**Published:** 2016-09-30

**Authors:** Gajendra J. Katuwal, Stefi A. Baum, Nathan D. Cahill, Chase C. Dougherty, Eli Evans, David W. Evans, Gregory J. Moore, Andrew M. Michael

**Affiliations:** ^1^Autism and Developmental Medicine Institute, Geisinger Health SystemDanville, PA, USA; ^2^Chester F. Carlson Center for Imaging Science, Rochester Institute of TechnologyRochester, NY, USA; ^3^Faculty of Science, University of ManitobaWinnipeg, MB, Canada; ^4^School of Mathematical Sciences, Rochester Institute of TechnologyRochester, NY, USA; ^5^Department of Psychology, Bucknell UniversityLewisburg, PA, USA; ^6^Institute for Advanced Application, Geisinger Health SystemDanville, PA, USA; ^7^Department of Radiology, Geisinger Health SystemDanville, PA, USA

**Keywords:** autism, brain imaging methods, brain volumes, SPM, FSL, Freesurfer, total intracranial volume, ABIDE

## Abstract

Previous studies applying automatic preprocessing methods on Structural Magnetic Resonance Imaging (sMRI) report inconsistent neuroanatomical abnormalities in Autism Spectrum Disorder (ASD). In this study we investigate inter-method differences as a possible cause behind these inconsistent findings. In particular, we focus on the estimation of the following brain volumes: gray matter (GM), white matter (WM), cerebrospinal fluid (CSF), and total intra cranial volume (TIV). T1-weighted sMRIs of 417 ASD subjects and 459 typically developing controls (TDC) from the ABIDE dataset were estimated using three popular preprocessing methods: SPM, FSL, and FreeSurfer (FS). Brain volumes estimated by the three methods were correlated but had significant inter-method differences; except TIV_SPM_ vs. TIV_FS_, all inter-method differences were significant. ASD vs. TDC group differences in all brain volume estimates were dependent on the method used. SPM showed that TIV, GM, and CSF volumes of ASD were larger than TDC with statistical significance, whereas FS and FSL did not show significant differences in any of the volumes; in some cases, the direction of the differences were opposite to SPM. When methods were compared with each other, they showed differential biases for autism, and several biases were larger than ASD vs. TDC differences of the respective methods. After manual inspection, we found inter-method segmentation mismatches in the cerebellum, sub-cortical structures, and inter-sulcal CSF. In addition, to validate automated TIV estimates we performed manual segmentation on a subset of subjects. Results indicate that SPM estimates are closest to manual segmentation, followed by FS while FSL estimates were significantly lower. In summary, we show that ASD vs. TDC brain volume differences are method dependent and that these inter-method discrepancies can contribute to inconsistent neuroimaging findings in general. We suggest cross-validation across methods and emphasize the need to develop better methods to increase the robustness of neuroimaging findings.

## Introduction

Structural magnetic resonance imaging (sMRI) is a powerful tool used to investigate the human brain *in vivo* and to find associations between brain morphometry and brain disorders. Although a large number of sMRI studies have been conducted, consistent sMRI markers for brain disorders are yet to be found (Chen et al., 2011). This can mainly be attributed to low statistical power of neuroimaging and neuroscience studies (Button et al., 2013). Inconsistent results may be due to differences in the demographics of data (Stanfield et al., 2008), image acquisition settings (Styner et al., 2002; Auzias et al., 2014), assumptions made on data and algorithms used (Eggert et al., 2012; Nordenskjöld et al., 2013; Fellhauer et al., 2015), and even machines used to process the data (Gronenschild et al., 2012).

With the increasing use of automated preprocessing methods in neuroimaging studies, the effect of inter-method variations in neuroimaging findings merit an investigation. Although automatic methods are more objective than manual methods, they possess method-specific bias and variance (Eggert et al., 2012; Nordenskjöld et al., 2013). The bias and variance across methods arise mainly due to method specific assumptions made on data, varying definition of brain structures, different image processing algorithms, varying sensitivity to imaging artifacts such as motion, and use of inconsistent *a priori* information such as brain templates. A number of previous studies have reported significant inter-method inconsistencies (Ren et al., 2005; Tsang et al., 2008; Eggert et al., 2012; Fellhauer et al., 2015; Hansen et al., 2015). Eggert et al. (2012) reported pronounced differences (11%) in mean segmented gray matter (GM) volumes from four standard segmentation algorithms: SPM8 New Segment, SPM8 VBM, FSL v.4.1.6 and FreeSurfer (FS) v.4.5. According to Hansen et al. (2015), compared to manual segmentation, FS 4.5 underestimated total intracranial volume (TIV) by 7 %. Similarly, differences in segmentation accuracies between FSL and SPM5 were reported by Tsang et al. (2008).

One important concern is that inconsistent results driven by inter-method variations can change the end results of a study and hence change the subsequent biological interpretation. Several previous studies have shown that the magnitude and even the direction of the effect size can be dependent on the method used. Boekel et al. (2015) performed a replication study on 17 brain structure-behavior correlations from five neuroimaging studies and were not able to replicate any of the correlations. A response paper by Muhlert and Ridgway (2015) pointed out that one of the reason the correlations could not be replicated is the methodological differences between SPM and FSL. Nordenskjöld et al. (2013), compared SPM8 and FS v.5.1.0 TIV estimates to reference TIV obtained from manual segmentation of proton density weighted images. They report that both SPM8 and FS overestimated TIV. In addition, SPM showed systematic bias associated with gender (systematic overestimation of TIV in females) and aging atrophy while FS showed bias for reference TIV (systematic overestimation of TIV for larger skull size). Notably, hippocampal volume showed different associations with education depending on which TIV measure (SPM or FS) was used for hippocampal volume normalization. When normalized with SPM TIV, there was no association between hippocampal volume and education, whereas when normalized with FS TIV, the association was significant. Similarly, Callaert et al. (2014) measured the effect of age on GM volume using four different methods: SPM8 Unified Segmentation, SPM8 New Segment, FSL v.4.1.5, and a method combining intensity based segmentation and atlas-to-image non-rigid registration. They found that the age specific effect changed with the different methods. Age related differences according to the Unified Segmentation and New Segment were significantly larger and smaller, respectively than other methods. Similarly, according to Rajagopalan et al. (2014), in ALS patients with frontotemporal dementia, FSL v.4.1.5 showed that the GM volume in motor region is significantly reduced, whereas SPM8 did not show any significant changes in GM. The above results suggest that inter-method discrepancies are a source of inconsistent findings in neuroimaging and that the choice of preprocessing method can affect end results. Thus, the effect of inter-method variations in neuroimaging results deserves a detailed investigation.

In this study we investigate inter-method discrepancies as a source of inconsistent neuroimaging findings in autism spectrum disorder (ASD). Brain anatomical findings in ASD compared to that of typically developing controls (TDC) have been highly inconsistent across studies (Amaral et al., 2008; Chen et al., 2011; Jumah et al., 2016; Katuwal et al., 2016). Recently a number of studies (Kucharsky Hiess et al., 2015; Valk et al., 2015; Haar et al., 2016; Riddle et al., 2016) have used the large multi-site (~1000 subjects, age 6–65 years) Autism Brain Imaging Data Exchange (ABIDE) (Di Martino et al., 2014) to investigate brain anatomical differences in ASD. Haar et al. (2016) using ABIDE did not replicate many of the previously reported anatomical abnormalities in ASD except significantly larger ventricular volumes, smaller corpus callosum volume (central segment only), and several cortical areas with increased thickness in the ASD group. One of the most replicated findings in ASD is that toddlers with ASD (age 2–4 years) on average have a larger head size than TDC (Courchesne et al., 2001, 2011; Carper et al., 2002; Campbell et al., 2014). However, several recent large studies (Raznahan et al., 2013; Zwaigenbaum et al., 2014) have shown that the there is no overall difference in head circumference between ASD and TDC over the first 3 years and the results of previous studies reporting large head sizes in ASD may be due to the bias in population norms. Campbell et al. (2014) have reported that abnormally rapid rate of brain growth during the first years of life seem to occur in a very small subgroup of ASD children.

Here we investigate if inter-method differences are a source of inconsistent neuroanatomical findings in ASD. We address this question by using global brain volume measures. We estimate gray matter (GM), white matter (WM), cerebrospinal fluid (CSF) volumes, and TIV of ASD and TDC subjects using the large ABIDE (Di Martino et al., 2014) dataset applying three widely used preprocessing methods: SPM, FSL, and FS. We answer the following three questions in this study. (1) How large are inter-method differences of brain volume estimates and how do they influence ASD vs. TDC group differences? (2) Do inter-method differences show differential bias toward diagnostic group (in this case ASD) and how does it compare with ASD vs. TDC differences? (3) What are potential reasons behind inter-method differences in brain volume estimation? We also compare TIV estimates of the three methods with ground truth obtained from manual segmentation on a small subset of subjects. We conclude with a discussion on potential causes of inter-method discrepancies and suggestions for alternative approaches.

## Methods

### Structural MRI and image processing

A total of 1112 sMRI scans were downloaded from the ABIDE consortium (http://fcon_1000.projects.nitrc.org/indi/abide/) representing imaging data from 17 different sites. Local Institutional Review Boards (IRB) approved the data acquisition in all participating sites. Consent to participate was obtained via IRB approval or explicit waiver. All ABIDE data are fully anonymized according to HIPAA guidelines. Each sMRI was visually inspected to detect significant motion and other artifacts. In total, 172 images with poor image quality and motion artifacts were discarded. An additional 64 subjects were discarded due to failure during segmentation by FS or FSL; none of the images failed during SPM segmentation. T1 weighted brain sMRIs from a total of 876 subjects from 15 sites were retained for volumetric analyses. Of the 876 subjects, 417 (367 males, 50 females) were ASD and 459 (382 males, 77 females) were TDCs. Subject demographics and behavioral measures of the 876 subjects used in this study are presented in Table 1. Scanner parameters used for each of the ABIDE sites are presented in Supplementary Table 1.

**Table 1:** **Subject demographics and behavioral (DB) measures**.

	**ASD**	**TDC**	**ASD vs. TDC *t*-test *P*-value**
N	417	459	
	*M* = 367, *F* = 50	*M* = 382, *F* = 77	
Age(years)	17.8 ± 8.9	17.7 ± 8.0	0.88
	(7–64)	(6.47–56.2)	
VIQ	104.6 ± 17.8	112.4 ± 12.9	3.1E-10^*^
PIQ	105.0 ± 16.7	108.1 ± 12.9	5.2E-3^*^
FIQ	105.4 ± 16.5	111.5 ± 12.1	4.3E-9^*^
ADOS	11.9 ± 3.7	NA	NA

*M, Male; F, Female; VIQ, Verbal IQ; PIQ, Performance IQ; FIQ, Full IQ; ADOS, Autism Diagnostic Observation Schedule*.

* *significant at 0.05*.

All sMRIs were preprocessed with SPM8 (Ashburner and Friston, 2005), FSL 5.0.4 (Jenkinson and Smith, 2001b), and FS 5.3.0 (Dale et al., 1999; Fischl et al., 1999). In order to minimize manual intervention and to make the study more objective and replicable, default parameters set by the respective toolboxes were used. Final results of the automatic segmentations were manually inspected and subjects with segmentation failures (64 in total) were discarded from the study.

#### Tissue segmentation

##### SPM

Tissue segmentation in SPM was performed using the New Segment tool (Ashburner et al., 2013) of SPM. New Segment utilizes a finite mixture model where mixture components are modeled as Gaussians. A modified Gaussian mixture model is updated by combining spatial information from a standard tissue probability map (TPM) and the intensity information of the input sMRI image (Ashburner and Friston, 2005). New Segment uses ICBM-452 T1 brain atlas (Mazziotta et al., 2001b) as the standard TPM. The posterior tissue probability after the last iteration results in GM, WM, and CSF segmented TPMs of the input sMRI. The value at a certain voxel of a TPM represents the probability of a certain tissue (GM, WM or CSF) belonging to that voxel. Further details on SPM tissue segmentation can be found at Ashburner et al. (2013).

##### FSL

Tissue classification in FSL was performed by *FAST* (Zhang et al., 2001). *FAST* is based on a hidden Markov random field (HMRF) model and uses the expectation maximization (EM) algorithm to find the maximum likelihood estimate of the model parameters. The HMRF model is a generalized version of the finite mixture model. The hidden variables specifying the identity of the mixture component (parametric distribution) of each observation (voxel intensity) are related by a Markov process in HMRF model unlike the finite mixture model where they are independent to each other. The HMRF model in *FAST* does not use standard TPM as *a priori*; instead it uses K-means segmentation to estimate the initial parameters of the tissue classes. Brain regions were extracted using the brain extraction tool (*BET*) (Smith, 2002) before tissue segmentation using *FAST*. Fractional intensity threshold (*f* option in *BET*) was kept at the default value of *f* = 0.5. Brain slices (from the *slicedir* directory) produced by FSL script *fslvbm_1_bet–b* were used to visually verify that brain regions were accurately extracted. Images for which brain extraction did not work properly with the default value of *f* = 0.5 were excluded from the study.

##### FS

Tissue segmentation in FS was performed by the *recon-all* preprocessing workflow (https://surfer.nmr.mgh.harvard.edu/fswiki/recon-all). *Recon-all* is a fully automated workflow that performs all the FS cortical reconstruction processes. *Recon-all* includes 31 processing stages beginning with motion correction, followed by non-uniform intensity normalization, Talairach transform computation, intensity normalization, skull stripping and ending with cortical parcellation steps. FS utilizes atlas-based segmentation whereby a new image is transformed to the standard MNI 305 atlas space (Collins et al., 1994). Similar to FSL, an HMRF model is utilized for segmentation in FS (Fischl et al., 2002). This model incorporates spatial information as well as intensity information and is updated in a Bayesian framework. Maximum *a posteriori* (MAP) estimate of the model parameters are used to compute the class labels of the voxels in an image. Further details can be found at (https://surfer.nmr.mgh.harvard.edu/fswiki/recon-all) and Bruce Fischl et al. (2002).

#### Brain volume calculation

##### SPM

Spm_get_volumes script was used to calculate the tissue volumes using c1, c2, and c3 images corresponding to native space tissue maps of GM, WM, and CSF, respectively. Native space volumes were selected in order to minimize volume changes due to spatial transformations. TIV was calculated as the sum of the GM, WM, and CSF volumes in the native space of the sMRI. This method of TIV calculation was performed according to SPM's recommendation (https://en.wikibooks.org/wiki/SPM/VBM) and has been utilized in several previous studies (Ridgway et al., 2011; Nordenskjöld et al., 2013).

##### FSL

Fslstats script was used to calculate the tissue volumes using partial volume maps (pve_0, pve_1, pve_2 images) produced by FAST (Zhang et al., 2001) as recommended by FSL (http://fsl.fmrib.ox.ac.uk/fsl/fslwiki/FAST#Tissue_Volume_Quantification). These partial volume maps are probablistic images of tissues in the native space. TIV was calculated using the SIENAX function of FSL as recommended by FSL (Smith et al., 2002) and the ENIGMA protocol (http://enigma.ini.usc.edu). SIENAX first strips out non-brain tissues using BET to extract the regions corresponding to the brain and the skull. After brain extraction, the skull image is affine registered to the MNI52 template (Mazziotta et al., 2001a) with a scaling factor between the subject's image and the standard space as the output. The above registration is carried out by the FSL's linear image registration tool—FLIRT (Jenkinson and Smith, 2001a). The scaling factor is computed as the determinant of the affine transformation matrix that registers the subject's image to the MNI152 template. Finally, the TIV of the subject's image is calculated by dividing the TIV of MNI152 template brain (1.847712 L) by the scaling factor (Mazziotta et al., 1995, 2001a,b).

##### FS

As recommended by FS, GM and WM volumes were extracted from *aseg.stats* file which is an output of the *recon-all* workflow (see https://surfer.nmr.mgh.harvard.edu/fswiki/MorphometryStats). FS does not output total CSF volume. In FS TIV is calculated by a technique similar to FSL (see http://surfer.nmr.mgh.harvard.edu/fswiki/eTIV). However, FSL uses both brain and skull whereas FS uses only the brain to guide the registration of a subject's image to a template. Estimated TIV (*eTIV*) is calculated by dividing the atlas mask volume from MNI305 template by the determinant of the affine transformation matrix (T) that maps the native space image into MNI space (Buckner et al., 2004). Talairach registration, the third step of *recon-all*, computes the affine transform *T* that transforms the original image to the MNI305 template (Evans et al., 1992).

### Statistical analysis

Statistical software package *R v3.2.0*. (http://www.R-project.org/) was used for all statistical analyses. Brain volume estimates from the three preprocessing methods were compared with each other to test inter-method differences and their biases for diagnostic group (ASD). All *p*-values reported in this study were corrected for multiple comparisons using false discovery rate (Benjamini and Hochberg, 1995) unless otherwise explicitly mentioned.

#### Inter-method differences in brain volumes estimation

Inter-method differences across all 876 subjects are summarized by the following statistics: mean volume difference, percentage difference, correlation coefficient, Cohen's d, and paired *t*-test *p*-value. Separate comparisons were performed for different volume types. Cohen's d (Cohen, 1988) was used as a measure of effect size. Cohen's d is the standardized difference between two means and is defined as *(mean_1_−mean_2_)/SD_pooled_* where *SD_pooled_* is the weighted average of the standard deviations of two groups. Paired *t*-tests were performed to test the statistical significance of the inter-method differences in brain volume estimates.

#### ASD vs. TDC inter-group differences in brain volumes

Autism Spectrum Disorder ASD vs. TDC brain volume differences were tested using independent two sample *t*-tests for each preprocessing method. In addition, ASD vs. TDC brain volume differences were tested after adjusting for the effects of age, sex, and site by fitting a linear mixed-model using *lmer4* package in R (Bates et al., 2014). As fixed effects, we entered diagnostic group (ASD/TDC), sex, age, and age^2^. As random effects, we included random intercepts and slopes for the effect of diagnostic group at each level of site. Fixed effect of diagnostic group is reported as the ASD vs. TDC group difference. The *p*-values were calculated using the Satterthwaite's approximated degrees of freedom (Satterthwaite, 1946) implemented in *lmertest* (Kuznetsova et al., 2013). In another separate experiment, in addition to age, sex, and site, the effect of full scale IQ (FIQ) was adjusted, where subjects with missing FIQ (*N* = 63) were excluded. For each method, separate models were built for different brain volume types.

#### Method bias for diagnostic group

The inter-method difference in brain volumes (△*y* = *y*_2_ − *y*_1_) were modeled by a linear mixed-model. The fixed and random effects of the model were the same as in the model described in previous section. Here, *y*_1_ and *y*_2_ are brain volume estimates from two different methods where *y*_1_ is considered as the reference value. The fixed effect of diagnostic group on △*y* (β_autism_) can be interpreted as the amount of brain volume by which method *y*_2_ systematically over/under estimates ASD subjects compared to TDC. Here our null hypothesis is that different methods do not have systematic differential bias to the diagnostic group. Our null hypothesis will be rejected when the fixed effect of diagnostic group β_autism_ is statistically significant. If β_autism_ is statistically significant, we will conclude that with reference to *y*_1_, method *y*_2_ has systematic biases for ASD or TDC subjects. Percentage bias of *y*_2_ for ASD (with reference to *y*_1_) was calculated as $\frac{\beta_{\text{autism}}}{\bar{y_{1}}}\times 100$, where $\bar{y_{1}}$ is the mean volume across all subjects according to *y*_1_. For each pair of methods, separate models were fitted for the different brain volumes. Multiple comparisons correction across different tissues was performed separately for each pair.

#### Experiments repeated with NYU data

Data used for the above experiments were acquired from multiple scanning sites and the effects of scanning site were adjusted by fitting a linear mixed-model with site as a random effect. This model does not capture the non-linear site effects. In order to completely eliminate the effects of site and to focus only on the differences due to methods, we repeated the above experiments using only the NYU site. NYU was selected because it had the largest number of subjects (71 ASD and 58 TDC) and all of its subjects had FIQ information. NYU results are presented as supplementary materials.

#### Manual segmentation for TIV

To determine which of the three methods examined were most accurate, we performed manual segmentation on 25 subjects from the NYU ABIDE site. Manual segmentation is a highly time consuming process and in order to reduce the workload manual segmentation was limited to only 25 subjects. These 25 subjects were identified by computing the inter-method difference of TIV between SPM, FSL, and FS, for each subject. These differences were then squared for all three methods and summed. From this calculation we identified the 25 subjects who exhibited the greatest total combined inter-method differences and selected them for manual segmentation. Manual segmentations were performed on every axial slice using ITKSNAP (Yushkevich et al., 2006).

For each slice, we segmented the intracranial region using ITKSNAP's polygon tool followed by fine corrections at the voxel level using a brush tool. Each MRI image took about 30 min by an experienced operator to perform the segmentation. The protocol detailed by Nordenskjöld et al. (2013) was followed to segment the images manually. The cranial cavity in each slice was outlined by tracing the dura, which includes all brain tissue and CSF inside the skull. All dural sinuses were included. Where dura was not visible, the cerebral contour was traced. The first slice where dura is visible from the top was the first segmented slice and the last slice containing cerebellum was the final segmented slice. The bilateral cavernous sinus and trigeminal cave were excluded from the segmentation. After manual segmentation, a quality check was performed on the images by a second operator to insure accuracy.

To determine which method corresponded most closely to our manual segmentation results, we computed the following statistics between each method's TIV estimate and manual segmentation TIV estimate: overall mean difference and standard deviation, Pearson's correlation and root mean squared (RMS) error.

## Results

### Estimated brain volumes and inter-method differences

**Figure 1:**
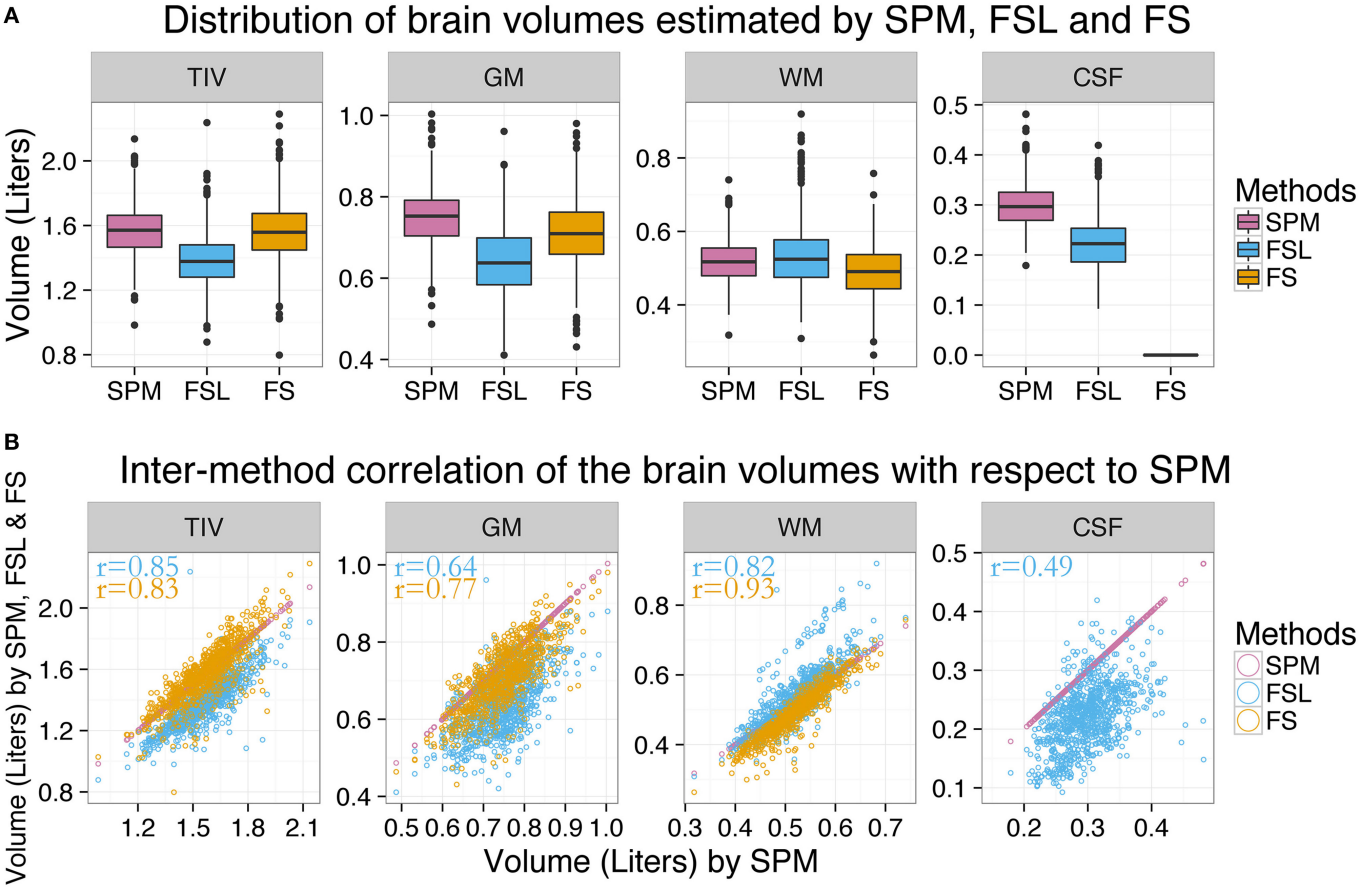
**Distribution of estimated brain volumes and inter-method differences**. **(A)** The distribution of brain volumes estimated by SPM (purple), FSL (blue) and FS (orange) are presented by boxplots and indicate significant inter-method differences. CSF_FS_ is not presented since FS does not output total CSF volume. **(B)** Volumes estimated by FSL and FS are plotted against the volumes estimated by SPM. The brain volume estimates from different methods had moderate agreement except for CSF.

Brain volumes estimated by SPM, FSL, and FS are presented in Table 2. Statistics of the inter-method differences (mean difference, correlation coefficient, Cohen's d, and paired *t*-test *p*-value) for all 876 subjects are presented in the shaded cells. The distributions of the estimated brain volumes are visualized as boxplots in Figure 1A. To visualize the inter-method distribution, the estimates from SPM, FSL, and FS are plotted against the estimates from SPM in Figure 1B.

**Table 2:** **Estimated brain volumes and inter-method differences**.

		**TIV**	**GM**	**WM**	**CSF**
SPM (L)		1.566 ± 0.15	0.748 ± 0.07	0.519 ± 0.06	0.299 ± 0.04
SPM vs. FSL	SPM–FSL mean diff. (ml)	183.4	106.0	−14.4	77.9
	Correlation Coefficient	0.851	0.639	0.821	0.494
	Cohen's d	1.20	1.41	−0.21	1.62
	Paired *t*-test *p*-value	<1E-100^**^	<1E-100^**^	7E-18^**^	<1E-100^**^
FSL (L)		1.383 ± 0.15	0.642 ± 0.08	0.533 ± 0.08	0.221 ± 0.05
FSL vs. FS	FSL–FS mean diff. (ml)	−177.4	−70.0	40.3	204.9
	Correlation Coefficient	0.829	0.745	0.808	NA
	Cohen's d	−1.05	−0.82	0.53	NA
	Paired *t*-test *p*-value	<1E-100^**^	<1E-100^**^	<1E-100^**^	NA
FS (L)		1.560 ± 0.18	0.708 ± 0.08	0.493 ± 0.07	NA
SPM vs. FS	SPM–FS mean diff. (ml)	6.1	40.1	25.9	
	Correlation Coefficient	0.830	0.767	0.934	NA
	Cohen's d	0.04	0.54	0.42	NA
	Paired *t*-test *p*-value	0.07	<1E-90^**^	<1E-100^**^	NA

Mean and standard deviation of the brain volumes estimated by SPM, FSL and FS are presented. Cells corresponding to CSF_FS_ are filled as “NA” since FS does not output total CSF volume. Inter-method differences and corresponding statistics are presented in shaded cells. SPM vs. FSL % difference was calculated as 100^*^$(\bar{SPM}-\;\bar{FSL})/\bar{FSL}$ and other comparisons were performed similarly. Correlation coefficient is used to measure the association between the brain volumes estimated by two different methods. Cohen's d is used to measure the effect size of the inter-method difference and paired t-test was used to check the statistical significance. Correlation coefficients show that methods agree on volume estimates but the Cohen's d and paired t-test p-values indicate significant inter-method differences.

** *significant at E-10*.

The brain volume estimates from different methods moderately agreed except for CSF (see Figure 1B). SPM vs. FSL correlation coefficients were 0.85, 0.64, 0.82, and 0.50 for TIV, GM, WM, and CSF volumes, respectively (see Table 2). Similarly, SPM vs. FS correlation coefficients were 0.83, 0.77, and 0.93 for TIV, GM, and WM volumes, respectively. FSL vs. FS correlation coefficients were 0.83, 0.75, and 0.81 for TIV, GM, and WM volumes, respectively. In summary, the inter-method correlation coefficients were high for TIV and WM, followed by GM and were the lowest for CSF.

Except WM, the average estimates of all the brain volumes by SPM were higher than that of FSL and FS; see purple boxplot in Figure 1A. FSL estimates of TIV were the lowest while FSL estimates of WM were the highest. Similarly, FS estimates of WM were the lowest. In summary, the following inter-method differences were observed: TIV_SPM_ (1.57 L) > TIV_FS_ (1.56 L) > TIV_FSL_ (1.38 L), GM_SPM_ (0.75 L) > GM_FS_ (0.71 L) > GM_FSL_ (0.64 L), WM_FSL_ (0.53 L) > WM_SPM_ (0.52 L) > WM_FS_ (0.49 L) and CSF_SPM_ (0.30 L) > CSF_FSL_ (0.22 L). Except TIV_SPM_ vs. TIV_FS_, all inter-method differences were statistically significant (*p* < 0.001; Cohen's d > 0.5 in 7 and *d* > 1 in 4 out of 10 comparisons).

The inter-method correlation coefficients within NYU subjects were slightly higher than that from using all subjects (see Supplementary Table 2). Inter-method differences were mostly similar to that of the experiment using all subjects.

### ASD vs. TDC inter-group differences is dependent on the method used

**Figure 2:**
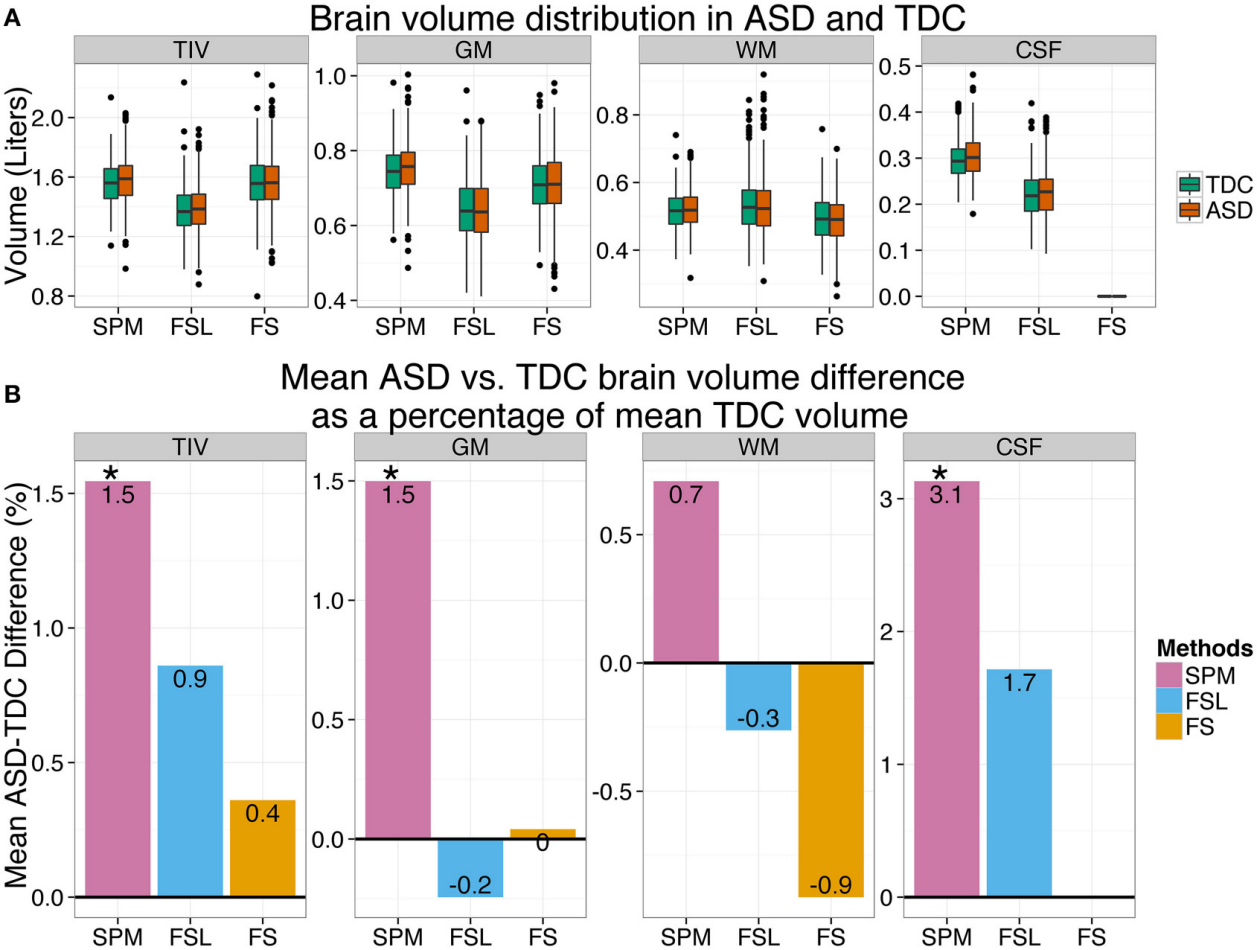
**ASD–TDC brain volume differences are preprocessing method dependent**. **(A)** The distribution of brain volumes estimated by SPM, FSL, and FS for ASD and TDC. **(B)** ASD vs. TDC brain volume difference as a percentage of mean TDC is presented as a bar plot for each method. ASD vs. TDC brain volume difference varied with methods which suggests that subsequent interpretations are highly dependent on the method of choice. ^*^Significant at 0.05.

Neuroimaging studies generally focus on investigating the average difference between two groups of subjects and hence it is important to probe how group differences are method dependent. Here we show how the inter-method differences in brain volume estimates can influence ASD vs. TDC inter-group differences. In other words, here we ask: “is inter-group difference dependent on the processing method used?” The mean ASD vs. TDC (ASD–TDC) group difference for TIV, GM, WM, and CSF according to SPM, FSL, and FS are presented in Table 3. The ASD vs. TDC distribution of the brain volume estimates are presented as boxplots in Figure 2A. The mean inter-group differences in percentage are presented as bar plots in Figure 2B.

**Table 3:** **ASD vs. TDC brain volume differences**.

	**TIV**			**GM**			**WM**			**CSF**		
	***diff (ml)***	***diff %***	***p-value***	***diff (ml)***	***diff %***	***p-value***	***diff (ml)***	***diff %***	***p-value***	***diff (ml)***	***diff %***	***p-value***
**RAW VOLUMES**												
SPM	24.0	1.53	0.019^*^	11.1	1.49	0.016^*^	3.7	0.71	0.33	9.2	3.08	0.001^*^
FSL	11.8	0.86	0.26	−1.6	−0.24	0.77	−1.4	−0.26	0.80	3.8	1.70	0.30
FS	5.6	0.36	0.65	0.3	0.04	0.96	−4.5	−0.92	0.33	NA	NA	NA
**ADJUSTED FOR AGE, SEX, AND SITE**												
SPM	20.8	1.34	0.04^*^	13.7	1.84	0.02^*^	3.7	0.71	0.34	5.9	1.99	0.011^*^
FSL	12.7	0.92	0.32	1.37	0.21	0.85	−0.1	−0.02	0.99	−1.6	−0.73	0.47
FS	8.3	0.53	0.60	3.11	0.44	0.64	−2.9	−0.59	0.57	NA	NA	NA
**ADJUSTED FOR AGE, SEX, SITE, AND FIQ**^§^												
SPM	28.1	1.81	0.001^*^	17.5	2.35	0.006^*^	6.2	1.22	0.13	6.5	2.22	0.006^*^
FSL	19.1	1.44	0.16	3.5	0.55	0.67	2.2	0.42	0.70	−1.4	0.62	0.55
FS	16.1	1.03	0.33	7.9	1.11	0.26	0.3	0.07	0.95	NA	NA	NA

§ 63 subjects with missing FIQ were excluded from the particular analysis. diff = Mean(ASD–TDC) difference. diff % = Mean (ASD–TDC) difference as a percentage of mean TDC volume. Cells corresponding to CSF_FS_ are filled as “NA” since FS does not output total CSF volume. Statistically significant differences are denoted by

* *for p < 0.05. ASD vs. TDC differences are dependent upon the method used and only in SPM, TIV, GM and CSF volumes in ASD were significantly larger than TDC*.

Results show that ASD vs. TDC differences are highly dependent upon the method used. According to SPM, ASD had 1.53% (*p* = 0.019) more TIV than TDC; see Table 3 and purple bar in Figure 2B. Whereas according to FS and FSL, ASD had only 0.36% (*p* = 0.65) and 0.86% (*p* = 0.26) more TIV than TDC, respectively (see yellow bar in Figure 2B). Similarly, according to SPM, ASD had 1.49% (*p* = 0.016) more GM than TDC. In contrast, FSL estimates show that ASD has 0.24% (*p* = 0.77) less GM than TDC. Whereas, according to FS, there was small difference in GM of ASD and TDC. Similar method dependent ASD vs. TDC differences in WM and CSF volume estimates were noted. Method dependence of ASD vs. TDC differences persisted even after the effects of age, sex, and site were removed (see Table 3). Similar results were observed even after removing the effects of FIQ where 63 subjects with missing FIQ were excluded from the analysis. We verified our results by removing the effects of site by using subjects from only one scanning site (NYU). Here again, ASD vs. TDC volume differences were dependent on the method used (see Supplementary Table 3).

### Differential bias of methods to the diagnostic group

Biases were pairwise calculated between methods using estimates from one method as the reference and are presented in Table 4. Compared to FSL, SPM showed statistically significant bias for ASD subjects in TIV (0.8%, *p* = 0.039), GM (1.9%, *p* = 0.007), WM (1%, *p* = 0.038), and CSF (3%, *p* = 0.006) volumes. Similarly, compared to FS, SPM showed statistically significant bias for ASD subjects in GM (1.4%, *p* = 0.004) and WM (1.5%, *p* = 0.004) volume estimates. We also noted that several method biases were larger than ASD vs. TDC differences according to the same methods. For example, with reference to FSL, SPM bias for ASD subjects in WM estimation was 7 ml, whereas, the inter-group difference in WM volumes according to SPM was 3.7 ml (see Table 3). In summary, with reference to FSL and FS, SPM showed positive bias for ASD subjects in multiple brain volumes. In other words, with reference to SPM, FSL, and FS showed negative bias in brain volume estimation of ASD subjects. Many of the biases were larger than ASD vs. TDC inter-group differences according to the respective methods. When we repeated these experiments using subjects from only one scanning site (NYU) we got similar results; see Supplementary Table 4 for details.

**Table 4:** **Differential bias for diagnostic group (ASD)**.

	**TIV**			**GM**			**WM**			**CSF**		
	***bias (ml)***	***bias %***	***beta p-value***	***bias (ml)***	***bias %***	***beta p-value***	***bias (ml)***	***bias %***	***beta p-value***	***bias (ml)***	***bias %***	***beta p-value***
SPM vs. FSL^#^	11	0.8	0.039^*^	12	1.9	0.003^*^	5	1.0	0.038^*^	7	3.0	0.006^*^
FSL vs. FS^#^	5	0.3	0.75	−1	−0.2	0.75	2	0.37	0.75	NA	NA	NA
SPM vs. FS^#^	13	0.8	0.180	10	1.4	0.004^*^	8	1.5	0.004^*^	NA	NA	NA

# *reference method bias (ml): brain volume (in ml) by which a method systematically overestimates in ASD subjects than in TDCs. % bias: the percentage of brain volume by which a method systematically overestimates in ASD subjects than in TDCs*.

* *significant at 0.05*.

### Manual segmentation

**Figure 3:**
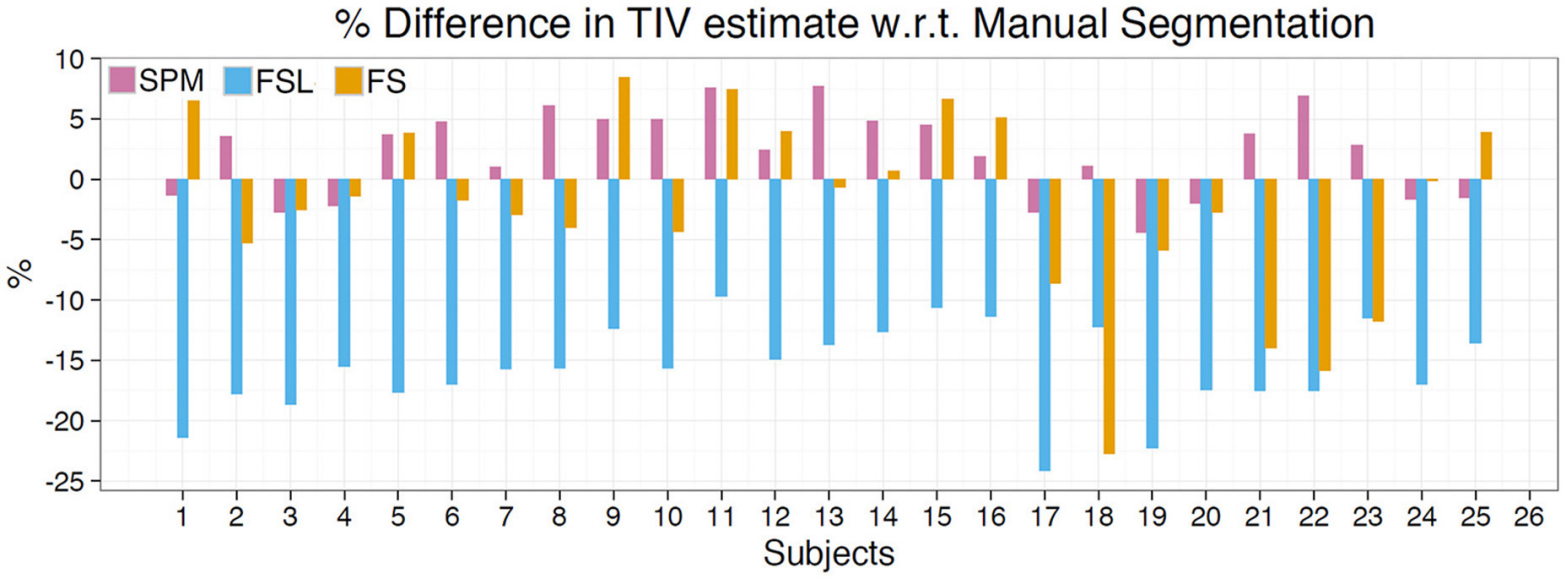
**TIV estimation difference compared to manual segmentation**. Depicts TIV estimation difference for each subject as a percentage of manual segmentation TIV. SPM overestimates for the majority of subjects while FS generally underestimates. FSL exhibited the greatest amount of difference and underestimated TIV for all subjects.

Results of this analysis are depicted in Table 5, Figure 3. The mean percentage difference between the methods and manual segmentation were as follows: SPM 2.16%, FS −2.31%, and FSL −16.4%. RMS error for these methods followed the same trend: SPM = 0.06, FS = 0.12, and FSL = 0.26. Pearson's correlation indicates that SPM exhibited the highest correlation with manual segmentation (*r* = 0.94) followed by FSL (*r* = 0.92) and FS (*r* = 0.84). The mean difference between manual segmentation and automated methods was greatest for FSL (mean difference = −251.89 ml) and SPM had the lowest mean difference (31.88 ml). In Figure 3 the percentage differences between estimated TIV and manual segmentation are presented for each of the 25 subjects. For all 25 subjects FSL under estimated the TIV with percentage differences in the range of −10 to −25%. Although SPM generally over estimated TIV and FSL generally underestimated TIV, percentage differences for these methods were in both positive and negative directions. SPM and FS percentage differences were in the range of −4 to 8% and −22 to 8%, respectively. The above statistics indicate that compared to FS and FSL, TIV estimates of SPM are closer to manual segmentation.

**Table 5:** **Comparison of Manual Segmentation TIV with Automated Methods**.

	**TIV Mean ± SD**	**Method–Manual difference (ml) Mean ± SD**	**Method–Manual % difference Mean ± SD**	**Pearson's correlation r (*p*-value)**	**RMS Error**
Manual	1.60 ± 0.16	NA	NA	NA	
SPM	1.64 ± 0.14	31.88 ± 55.67	2.16 ± 3.59	0.94 (3E-12)	0.06
FSL	1.35 ± 0.15	−251.89±60.97	−15.73±3.64	0.92 (4E-11)	0.26
FS	1.57 ± 0.22	−34.47±119.34	−2.31±7.77	0.84 (1.6E-7)	0.12

*Method–Manual % differences were calculated as a percentage of Manual TIV. Multiple statistical measures indicate that SPM estimates are closest to manual segmentation, followed by FS and FSL*.

## Discussion

In this work we investigated discrepancies in brain volumes (TIV, GM, WM, and CSF) estimated by three different preprocessing methods (SPM, FSL, and FS). Brain volume estimates between the methods had significant correlation, but the absolute values of brain volume estimates were significantly different between methods. In other words, there was significant method specific biases while estimating brain volumes. When the methods were compared pair-wise, significant differential biases for the diagnostic group (ASD) were revealed, and most biases were larger than ASD vs. TDC differences according to the respective methods. These biases had an influence on ASD vs. TDC brain volume differences and our results indicate that ASD vs. TDC brain volume differences were dependent on the method used to estimate the brain volumes. Below we compare our results with previous findings and discuss potential causes of brain tissue volume discrepancies by investigating segmentation disagreements at anatomical locations. We further provide discussions on inter-method discrepancies at a conceptual level. Finally, we discuss methods for minimizing inter-method discrepancies.

### ASD vs. TDC inter-group differences dependent on the method used

In this study we found that ASD vs. TDC inter-group differences in brain volumes are dependent upon the method used. This dependency was evident in all brain volume types. For example, SPM showed 1.53% more TIV in ASD compared to TDC with statistical significance. Whereas FS showed that ASD had only 0.36% more TIV compared to TDC and the difference was not statistically significant. In other words, a research study using SPM to estimate TIV will report statistically significant TIV_ASD_ > TIV_TDC_ but a different study using FS on the same data will not report statistically significant difference. Similarly, ASD had 1.49% more GM than TDC according to SPM, and the difference was statistically significant. Whereas according to FSL, ASD had 0.24 % less GM than TDC. This means— a study using SPM would report larger GM volumes in ASD whereas a study using FSL would report smaller GM volumes in ASD. These results show that the magnitude and even the direction of the effect under investigation is dependent on the method used, and previous studies have reported similar findings. For example, Callaert et al. (2014) reported that the age effect on GM volume was significantly dependent upon the method used to estimate the GM volume. Similarly, Nordenskjöld et al. (2013) reported that hippocampal volume showed different associations with education depending on which TIV measure (SPM or FS) was used for hippocampal volume normalization. Rajagopalan et al. (2014) found significant GM reduction in the motor region of the brain of ALS patients using SPM but could not replicate the finding using FSL. These results demonstrate that the choice of a preprocessing method used to estimate brain volumes can have a significant effect on the end results of a study.

### Comparison of TIV estimates with manual segmentation

According to the results of manual segmentation on a subset of subjects, SPM most closely approximates TIV estimates obtained via manual segmentation, followed by FS, and then FSL. Although the overall mean difference in FS was not very high, compared to SPM there was greater variability in TIV estimates of FS. These results suggest that of the three automated methods evaluated SPM estimates are closest to manual segmentation. It is important to note that default parameters were utilized for all toolboxes and therefore we cannot extrapolate these results to situations in which user selected parameters are chosen. The TIV estimates for SPM generally overestimated for each subject compared to manual segmentation while FS generally underestimated. FSL by contrast underestimated for all 25 subjects examined (see Figure 3). For all three methods the mean TIV estimate as well as standard deviation of the manual segmentation closely matched those of the full sample (see Table 2).

Discrepancies between TIV calculated via automated methods and manual methods can be partly attributed to differing definitions of TIV for each toolbox. For example, in SPM, TIV was calculated as the summation of the tissue volumes: GM, WM, and CSF (https://en.wikibooks.org/wiki/SPM/VBM). Whereas in FSL and FS, TIV was estimated using the atlas scaling factor technique (Buckner et al., 2004). Even though FSL and FS use similar techniques there are some methodological differences. For example, FSL uses MNI152 brain template whereas FS uses MNI305 brain template. Moreover, FSL uses both brain and skull whereas FS uses only the brain to guide the registration of a subject's image to the brain template. The differing definitions coupled with methodological differences can explain the errors in TIV.

With this comparison we provide evidence that compared to manual segmentation, TIV estimates of automated methods are different. In addition, with this validation we also provide evidence that TIV estimates of SPM are closer to manual segmentation than FS and FSL. This result further indicates that the significantly higher TIV in ASD as estimated by SPM is more accurate than the ASD vs. TDC comparisons of FS and FSL (see Table 2). We limited our manual segmentation validation just to TIV as TIV is the measure that can be easily estimated with visual inspection. Manual segmentation of GM, WM, and CSF regions is much harder and we did not perform validation of those regions. But from our TIV analysis we expect that automated estimates of those regions will be different from manual estimates.

### Comparison of ASD vs. TDC brain volume differences with previous studies

Due to various heterogeneous neuroimaging findings in ASD our comparisons here are limited only to meta analytic studies (Redcay and Courchesne, 2005; Stanfield et al., 2008; Radua et al., 2011; Via et al., 2011) and studies that used the ABIDE dataset (Kucharsky Hiess et al., 2015; Haar et al., 2016; Riddle et al., 2016). It should be noted that the exclusion criteria and the number of subjects included in the studies that used ABIDE data were not consistent across studies. In our study, TIV in ASD was larger than TDC according to all methods, however, the difference was statistically significant only according to SPM. Riddle et al. (2016) using SPM8 VBM also report greater TIV in ASD (1.58%) with statistical significance. Kucharsky Hiess et al. (2015) used a different toolbox (ART Brainwash, www.nitrc.org/projects/art) and reported higher TIV in ASD (1.73%) with statistical significance. Haar et al. (2016) using FS found TIV to be higher only in two of the 18 sites they used and when these two sites were removed (26 subjects) overall ASD vs. TDC difference was not statistically significant and this result is similar to our FS results. Further, Redcay and Courchesne (2005) and Stanfield et al. (2008) have also reported similar results. Interestingly, 1.53% more TIV in ASD reported by SPM in our study is very close to an estimate (1.534%) predicted by a model proposed by Redcay and Courchesne (2005). This model prediction was for ASD and TDC subjects at age 17.75 years which is the mean age of the subjects used in our study. Kucharsky Hiess et al. (2015) have also reported similar prediction.

In our study GM volume in ASD was greater than TDC (1.5%) with statistical significance according to SPM. An ABIDE study by Riddle et al. (2016) using SPM8 VBM also report a similar result (1.58% more in ASD). However, in our study GM volume in ASD was slightly smaller according to FSL (0.2%) without statistical significance and there was little difference in GM volume according to FS. Haar et al. (2016) report slightly larger GM volume in ASD but without statistical significance, using both FS (*d* = 0.01 in cortical GM; *d* = 0.13 in cerebellar GM) and FSL (*d* = 0.18). A large meta-analytic study by Via et al. (2011) report no GM volume differences (*d* = 0.006) between ASD and TDC. In summary, studies using SPM tend to report slightly larger GM volume in ASD but results of studies using FSL and FS are inconsistent.

In our study WM volume in ASD was slightly greater than in TDC (0.7%) without statistical significance according to SPM. Riddle et al. (2016) also using SPM report similar results (0.67% more in ASD). Similarly, Haar et al. (2016) report slightly larger WM volume in ASD but without statistical significance, using both FSL (*d* = 0.03) and FS (*d* = 0.13 in cortical WM; *d* = 0.04 in cerebellar WM). However, in our study, WM volume in ASD was slightly smaller according to both FSL (0.3%) and FS (0.9%) without statistical significance. A large meta-analytic study by Radua et al. (2011) report slightly smaller WM volume (*d* = 0.006) in ASD. In summary, studies using SPM tend to report slightly larger WM volume in ASD but results are inconsistent in studies that used FSL or FS.

In our study CSF volume was larger in ASD according to SPM (3.1%) with statistical significance. Similarly Lin et al. (2015) using SPM8 New Segment also found greater CSF volume in ASD (4.75%) with statistical significance. Riddle et al. (2016) using SPM8 VBM also report 1.54% more CSF in ASD but with no statistical significance. Haar et al. (2016) using FSL also report slightly larger CSF volume in ASD (*d* = 0.15) without statistical significance and this is similar to our FSL finding; 1.7% greater in ASD without statistical significance. In summary, our result agrees with previous finding of greater CSF volume in ASD and that the magnitude of the difference is method dependent.

### Methods have different biases for diagnostic group, and many of them are larger than inter-group differences

We found that methods have systematic differential biases for diagnostic group (ASD) and several biases were larger than ASD vs. TDC differences according to the respective methods. In other words, inherent systematic bias of a method to a variable of interest (ASD) is larger than the actual effect of the variable (brain volume difference due to ASD). The differential biases shown by the methods for ASD explains the method dependent ASD vs. TDC group difference in brain volumes presented in ASD vs. TDC inter-group differences in brain volumes. With reference to FSL and FS, SPM showed positive bias for ASD subjects in multiple brain volumes. From a different perspective or considering SPM as the reference method, it can be said that FSL and FS showed negative bias for ASD subjects. In other words, with reference to SPM, FSL and FS systematically underestimated brain volumes in ASD subjects compared to TDC. This might be one reason why SPM shows greater brain volumes in ASD compared to TDC while FSL and FS do not. To conclude which method captures the true ASD vs. TDC difference further investigation using ground truth data is necessary.

Similar results have been reported in previous studies (Nordenskjöld et al., 2013), where it was reported that SPM showed bias associated with gender and atrophy while FS showed bias dependent on skull size. In summary, the above results indicate that systematic differential biases of a preprocessing method can be assigned as brain volume differences due to autism thus leading to incorrect findings.

### Locations of the inter-method segmentation discrepancies

**Figure 4:**
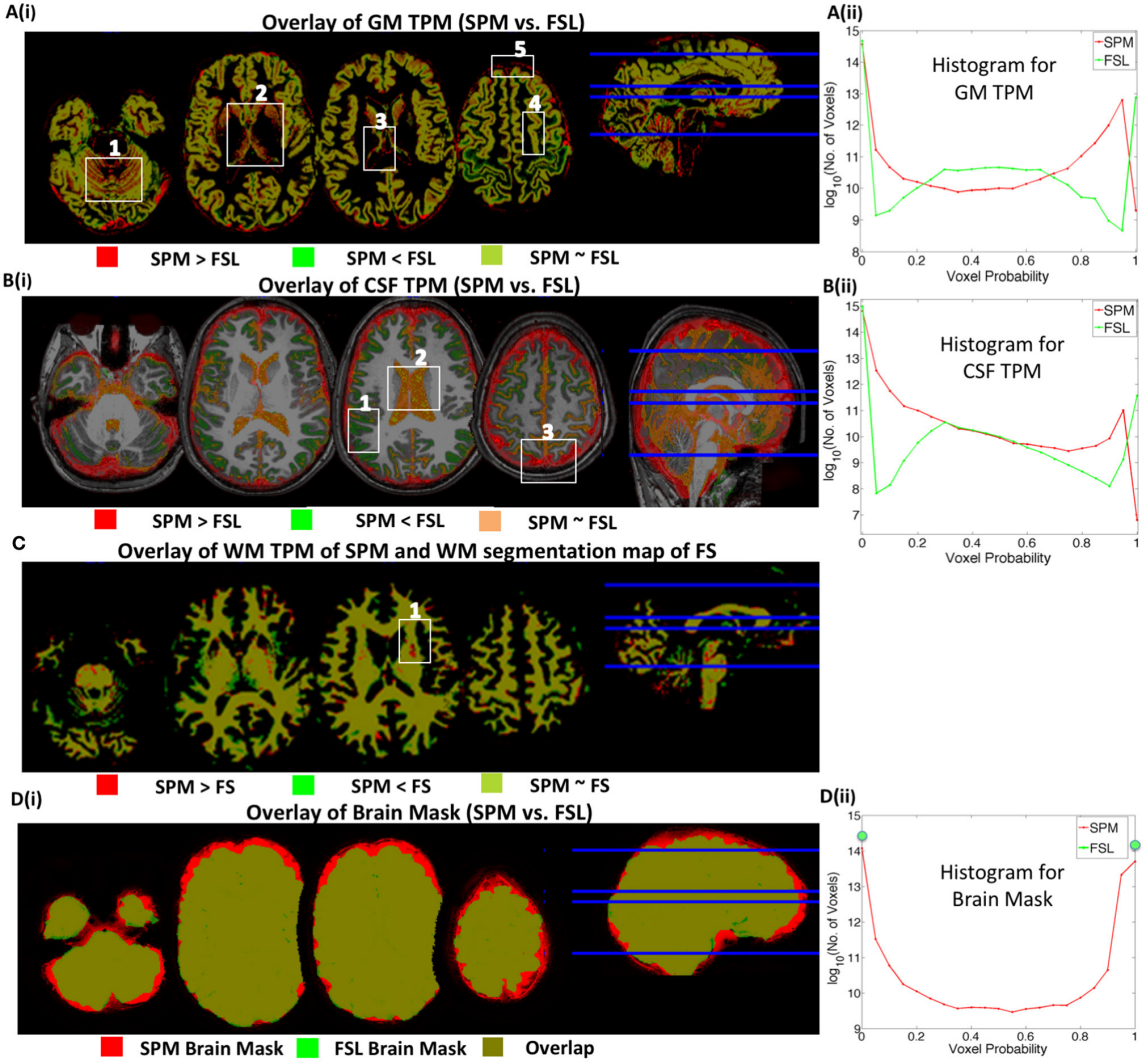
**Inter-method segmentation comparison**. Tissue Probability Maps (TPMs) from different methods are overlaid on one another. Red/green represents the voxels where only one TPM has non-zero probability value. Yellowish green or orange represents overlapping regions. **(A)** SPM vs. FSL GM segmentation, **(B)** SPM vs. FSL CSF segmentation, **(C)** SPM vs. FS WM segmentation and **(D)** SPM vs. FSL full brain map (GM+WM+CSF). **(Aii,Bii)** are histograms of voxel probability values in GM and CSF TPMs, respectively. *Although TPMs of different methods predominantly overlap, there are mismatching regions/voxel values of segmentation that contribute to inter-method differences in brain volumes estimates*.

To identify the locations of inter-method segmentation discrepancies, 20 subjects were randomly chosen and their tissue probability maps (TPM) were individually inspected using MRIcron (http://people.cas.sc.edu/rorden/mricron/index.html). For each tissue type, a subject with the most common segmentation discrepancy was chosen and these discrepancies are presented in Figure 4 where TPMs of different methods are overlaid using MRIcron.

#### Overestimation of GM by SPM compared to FSL and FS

In Figure 4Ai, red represents the regions where voxel probabilities in SPM TPM for GM are higher than that of FSL TPM; green, vice-versa and yellowish-green represents regions where voxel probabilities from both methods are similar. Figure 4Aii compares the histograms of the voxel probabilities in GM TPMs produced by SPM and FSL.

Our results showed that SPM overestimated GM volume compared to FSL in the following four main brain regions. (1) *Cerebellum:* FSL under segments GM (less GM volume or less GM voxel probability) in the cerebellum–see red regions in box 1 of Figure 4Ai. (2) *Subcortical Structures:* The proportion of GM (compared to WM) according to SPM is greater in subcortical structures–see red regions in box 2 in Figure 4Ai. Probability values in subcortical voxels are generally greater than 0.8 in GM TPM for SPM. This accounts for the higher red curve for voxel probabilities greater than 0.8 in the histogram of voxel probability presented in Figure 4Aii. In FSL, however, subcortical voxel probability values are in the 0.3–0.6 range, which accounts for the higher green curve in the 0.3–0.6 range. (3) *Boundaries of GM and other Structures:* SPM assigns regions close to the GM boundaries of different brain structures as GM indicated by red lines in box 3 and box 5 of Figure 4Ai. (4) *Inter-sulcal CSF:* SPM segments some inter-sulcal CSF as GM–indicated by red regions in box 4. The overestimation of GM in these brain regions by SPM explains the higher GM estimates by SPM.

#### Low correlation in CSF volume estimates by SPM and FSL

SPM and FSL produced similar ventricular CSF segmentations; overlap or agreement is presented in orange, box 2 in Figure 4Bi. However, the probability values in ventricular voxels are in the 0.95–0.99 range in CSF TPM of SPM, while probability values are exactly 1 in CSF TPM of FSL. This accounts for the shift of the FSL (green) curve to the right in the 0.9–1 range in Figure 4Bii. Discrepancies in non-ventricular CSF estimates were mainly from following three brain regions. (1) *Brain Boundary:* The estimation of CSF surrounding the brain, indicated by the red regions in box 3 and in other brain slices of Figure 4Bi was higher for SPM. This accounts for the higher SPM (red) curve in the 0.7–0.99 range in the histogram of CSF. This may be due to the fact that the *a priori* CSF TPM used by SPM (in New Segment) during segmentation has a thick layer of CSF at the boundary of the brain. (2) *Inter-sulcal CSF:* FSL segments greater CSF compared to SPM in inter-sulcal regions (see green regions in Box1 of Figure 4Bi), where CSF TPMs of SPM and FSL have probability values in the ranges of 0–0.3 and 0.3–0.7, respectively. This introduces higher SPM (red) curve in the 0–0.3 range in Figure 4Bii.

Our results indicate that the segmentation discrepancy in non-ventricular CSF segmentation is the primary cause of the low inter-method correlation in CSF volumes. Misclassification of bone/air as CSF or vice-versa can be another major source of discrepancy. T1-weighted images provide a reasonable amount of contrast between GM (dark gray), WM (lighter gray) and CSF (black). However, dense bone and air also appear dark like CSF. This makes the segmentation of CSF challenging, especially at the sulcal regions since it is difficult to distinguish between the inner skull and sulcal CSF. Accuracy in CSF segmentation can be improved by augmenting information from the T2-weighted image as it provides additional contrast between CSF (bright) and brain tissue (dark).

#### Underestimation of WM by FS compared to SPM

The WM volumes estimated by FS were the lowest in general. The final results of surface reconstruction and parcellation produced by *recon-all* were used to report WM segmentation by FS. Our study indicates that FS produces a considerable number of areas where WM is misclassified as non-WM (red dots in box 1 of Figure 4C). The misclassified areas were primarily due to WM hypo-intensities misclassified as GM or partial voluming in which WM + GM voxels look like non-WM and are segmented as non-WM. WM hypo-intensities have values much lower than the average WM intensity. Although recon-all automatically adds the volume of WM hypo-intensities to the total WM volume, our results indicate that it cannot still identify all the hypo intensities and the WM segmentations of FS require significant manual editing.

#### Brain mask (SPM vs. FSL)

Brain masks in SPM and FSL presented in Figure 4Di were created by the summation of GM, WM, and CSF TPMs. The brain mask of SPM (red) is larger than that of FSL (green). This is mainly due to the overestimation of CSF surrounding the brain by SPM compared to FSL. In the FSL brain mask, voxel probabilities have only two values, zero or one, while the voxel probabilities in SPM brain masks are continuous in the 0–1 range (see Figure 4Dii). In FAST of FSL, the HMRF model used for classification has only three components (GM, WM, and CSF); hence, the summation of these TPMs add up to one in the brain regions. In SPM, however, the Gaussian mixture model uses a mixture of six Gaussian components for GM, WM, CSF, bone, soft tissue, and air/background; but the brain mask was created by summing only three mixture components: GM, WM and CSF. Therefore, the voxel probabilities in the brain mask of SPM are in the 0–1 range.

### Sources of the inter-method discrepancies in tissue segmentation

Inter-method segmentation discrepancies in different locations of the brain were shown in the previous section. This section discusses the possible reasons behind the inter-method segmentation discrepancies at a conceptual level. Inter-method differences in tissue segmentation can be mainly attributed to differences in two factors: (1) method dependent differences in the brain template and method dependent differences in the spatial normalization process.

#### Brain templates

A brain template or atlas is an anatomical representation of a brain. It is a pre-segmented standard brain image generated from a single subject or a cohort of subjects. A brain template is generally used as an a priori to guide tissue classification. The different preprocessing methods used for tissue segmentation in this study utilize different standard brain templates for prior spatial information of the brain structures. For example, New Segment of SPM uses ICBM-452 T1 brain atlas (Mazziotta et al., 2001b) and FS uses MNI 305 (Collins et al., 1994) brain atlas. Whereas FAST of FSL does not use brain atlas but utilizes HMRF model to encode spatial information through contextual constraints of neighboring pixels in an image (Zhang et al., 2001). The differences among brain templates can arise primarily from two sources. First, the definition of brain structures can vary among the brain templates. Second, the brain templates are created from different cohorts of subjects scanned under different scanners and hence have different biases for subject demographics, image quality, and scanner settings. The inter-method discrepancies in brain structures segmentation can be minimized with the use population specific brain templates (Tang et al., 2010; Mandal et al., 2012).

#### Spatial normalization

Spatial normalization is the process whereby individual sMRIs are registered to the common anatomical space defined by the standard brain template. Spatial normalization conceptually consists of two elements: image representation and transformation. The differences due to spatial normalization starts from the choice in the mathematical representation of an image, i.e., how an image is mathematically represented to be used in subsequent mathematical operations. For image representation, several assumptions are made about the properties of the images, and these assumptions vary with the preprocessing methods. The effect of the differences in these assumptions propagate further and are finally evident with the discrepant segmentation results. Similarly, differences arise also from the choice of the transformation applied to the individual images to register it to a space defined by a standard template. In addition, different spatial normalization techniques behave differently with different image acquisition parameters, motion artifacts, and imaging artifacts such as bias field and intensity inhomogeneity. This adds further discrepancy to the segmentations.

### Implications for future neuroimaging studies

Our findings have important implications for the ongoing search for neuroimaging biomarkers in ASD and other brain disorders. Inconsistencies across previous studies and lack of evidence for brain biomarkers in ASD may in part be a result of failure to account for the issues we have raised in this study. Inter-method differences can also impact brain connectome studies (both structural and functional) where total brain volume is often used as a covariate. To reduce the impact of inter-method differences, we suggest the following directions that need further investigation.

#### Cross-validation of findings

Results of our study and of many previous studies (Eggert et al., 2012; Nordenskjöld et al., 2013; Callaert et al., 2014; Rajagopalan et al., 2014) have demonstrated the method dependence of neuroimaging results. Each method has its own strengths and weakness, and there is no general agreement on which method is optimal. Therefore, we suggest using multiple methods to segment brain images to cross validate results across methods. In addition, a multi-variate classifier can be trained on the outputs of several methods to improve the overall segmentation results. A simple classifier would be a majority voting system where the final decision is made based on the majority votes. For example, when SPM, FSL, and FS are used for binary tissue segmentation and if SPM and FSL labels a voxel as CSF whereas FS labels it as GM, then the multivariate system would label the voxel as CSF.

#### Development of better methods

The present preprocessing schemes apply spatial transformations that may introduce errors in tissue segmentations and are one of the major causes of inter-method differences. Machine learning based methods can be a way to perform segmentation with minimal preprocessing, and among them, deep-learning methods have proven to be more promising. Recently, a few studies have successfully performed the segmentation of brain structures from MRI using deep learning. De Brébisson and Montana (2015) have reported competitive accuracy for segmentation of cortical and sub-cortical structures from MRIs without performing any non-linear registration. Similarly, (Kim et al., 2013; Lai, 2015) have reported successful segmentation of hippocampus using deep learning.

#### Focus on combining image types in addition to methods

Accurate segmentations may not be possible using a single type of sMRI since it may not have sufficient contrast to discriminate the boundaries of brain structures. For example, T1-weighted sMRIs do not have enough information to distinguish between brain structures. It is difficult to segment CSF surrounding the brain region using T1-weighted MRIs since skull bone as well as CSF appear dark in the T1-weighted images. A very low inter-method correlation of 0.5 in CSF volume estimates obtained in this study demonstrates the difficulty. Whereas CSF appears bright and skull bone appears dark in T2-weighted MRI and this is very helpful for the CSF segmentation. Segmentation accuracy of CSF as well as other brain structures can be improved if information from multiple types of MRIs are used together. Numerous segmentation algorithms that use multiple types (MR sequences) of MRI are currently available and a collection of these algorithms with their segmentation accuracies can be found at http://mrbrains13.isi.uu.nl/.

### Limitations of our study

This study has several limitations. First, a multi-site data was used and many studies have shown image acquisition settings affect the quantification of brain morphometry from MRI using automated processing tools (Styner et al., 2002; Auzias et al., 2014). We removed the site effects by using a linear mixed-effect model with site as a random effect. In addition, we repeated our experiments in subjects scanned in only one site. Second, motion artifacts have considerable effect on automatically extracted MRI measures (Blumenthal et al., 2002; Brown et al., 2010). We performed strict quality check and removed 236 (out of 1112) images with motion and other artifacts. However, the image quality assessment itself is a subjective process and we acknowledge that little motion and other artifacts present in the deemed good quality images might have an effect in the results. Further, motion and other artifacts may have influenced the estimated brain volumes differently across the three methods, if this is true this again shows inconsistency across methods. Third, the definitions of GM, WM, CSF, and TIV differ among the methods. For example, when we are comparing CSF from SPM and CSF from FSL, we are not comparing exact same voxels. This limitation itself is one of the messages we are trying to convey in this paper. Here we are demonstrating that the results are method dependent because even the definition of brain structures are different across methods.

## Conclusions

We demonstrate that ASD vs. TDC group differences in brain volumes are method dependent. According to SPM, ASD brain volumes were higher than TDC with statistical significance but according to FSL and FS the differences were not significant. Further, validation via manual segmentation indicates that SPM provides TIV estimates closest to manual segmentation followed by FS and then FSL. Inter-method brain volume differences can be attributed to varying definitions of brain structures, use of different templates, differences in image processing algorithms, and the varying effects of imaging artifacts and acquisition settings. We suggest that research studies should cross-validate findings across multiple methods before providing biological interpretations. To our knowledge current studies do not account for the method dependency of results. Accounting for methodological differences will be an important step in increasing the reliability and consistency of future neuroimaging findings of autism and other brain disorders, leading to a greater likelihood of establishing valid and reliable neuroimaging biomarkers. We also emphasize that future work is needed to investigate the reasons behind inter-method discrepancies and the need to develop better methods.

## Author contributions

Contributions to this work are as follows: AM is the PI of the project. AM, GK, SB, and NC designed the study. GM, DE, CD, and EE contributed to the design of the study. EE downloaded the data and manually checked the quality of each MR image. GK, EE, and CD preprocessed MRI data and GK and AM performed the analyses. SB, NC, GM, DE, EE, and CD contributed to the analyses of the study. GK, AM, and SB drafted the manuscript. All authors contributed to the editing of the manuscript. All authors read and approved the manuscript.

## Funding

This project was internally funded by Geisinger Health System.

### Conflict of interest statement

The authors declare that the research was conducted in the absence of any commercial or financial relationships that could be construed as a potential conflict of interest.
