# Supplementary material for: Inter-Method Discrepancies in Brain Volume Estimation May Drive Inconsistent Findings in Autism
Source: Front Neurosci. 2016 Sep 30;10:439. doi: 10.3389/fnins.2016.00439 (PMC5043189; doi:10.3389/fnins.2016.00439)
Supplement: Supplementary file 1 [file Table1.DOCX]

**Supplementary Table 1: Scan parameters in data collection sites**

| **Site** | **Scanner Model** | **Scan**  **Time (min)** | **Voxel**  **Size**  **(mm)** | **Orientation** | **Slices**  **Per**  **Slab** | **Slice**  **Thickness**  **(mm)** | **TR**  **(sec)** | **TE**  **(sec)** | **Echo**  **Spacing**  **(sec)** |
| --- | --- | --- | --- | --- | --- | --- | --- | --- | --- |
| CALTECH | SMTS | 3:43 | 1 | Sagittal | 176 | 1 | 1590 | 2.73 | 7.9 |
| CMU | SMVS | 4:21 | 1 | Sagittal | 176 | 1 | 1870 | 2.48 | 7.4 |
| KKI | PA | 8 | 1 | Coronal | 200 | 1 |  |  |  |
| KUL | PI |  | 0.97 | Coronal | 182 |  |  | 4.6 |  |
| MPG | SMVS | 3:41 | 1 | Sagittal | 160 | 1 | 1800 | 3.06 | 7.3 |
| NYU | SMAS | 8:07 | 1.3 | Sagittal | 128 | 1.33 | 2530 | 3.25 | 7.4 |
| OHSU | SMTS | 9:14 | 1 | Sagittal | 160 | 1.1 | 2300 | 3.58 | 8.2 |
| OLIN | SMAS | 7:37 | 1 | Sagittal | 176 | 1 | 2500 | 2.74 | 7 |
| PITT | SMAS | 8:59 | 1 | Sagittal | 176 | 1.05 | 2100 | 3.93 | 9.4 |
| SBL | PI | 04:11 | 1 | Transverse | 170 | 1 |  |  |  |
| SJH | PA | 7:29 | 1 | Sagittal | 160 |  | 8.5 | 3.9 |  |
| UCLA | SMTS | 9:14 | 1 | Sagittal | 160 | 1.2 | 2300 | 2.84 | 6.6 |
| UM | GS |  |  | Axial | 40 | 3 | 250 | 5.7 |  |
| USM | SMTS | 9:14 | 1 | Sagittal | 160 | 1.2 | 2300 | 2.91 | 6.8 |
| YALE | SMTS | 8:34 | 1 | Sagittal | 176 | 1 | 1230 | 1.73 | 5.4 |

**SMTS:** SIEMENS MAGNETOM TrioTim syngo

**SMVS:** SIEMENS MAGNETOM Verio syngo

**PA:** PHILIPS ACHIEVA

**PI:** PHILIPS INTERA

**GS:** GE SignaÊ

**CALTECH:** California Institute of Technology

**CMU:** Carnegie Mellon University

**KKI:** Kennedy Krieger Institute

**KUL:** University of Leuven

**MPG:** Ludwig Maximilian University Munich

**NYU:** New York University

**OHSU:** Oregon Health and Science University

**OLIN:** Olin, Institute of Living at Hartford Hospital

**PITT:** University of Pittsburgh

**SBL:** Social Brain Lab

**SJH:** Trinity Center for Health Sciences

**UCLA:** University of California, Los Angeles

**UM:** University of Michigan

**USM:** University of Utah School of Medicine

**YALE:** Yale University
