# Supplementary material for: Inter-Method Discrepancies in Brain Volume Estimation May Drive Inconsistent Findings in Autism
Source: Front Neurosci. 2016 Sep 30;10:439. doi: 10.3389/fnins.2016.00439 (PMC5043189; doi:10.3389/fnins.2016.00439)
Supplement: Supplementary file 2 [file Table2.DOCX]

**Supplementary Table 2: Inter-method differences in NYU**

|  | | **TIV** | **GM** | **WM** | **CSF** |
| --- | --- | --- | --- | --- | --- |
| **SPM (L)** | | 1.517 ± 0.16 | 0.730 ± 0.07 | 0.507 ± 0.06 | 0.280 ± 0.04 |
| **SPM vs. FSL** | SPM – FSL *mean diff. (ml)* | 197.4 | 65.0 | 15.22 | 126.6 |
|  | *Correlation Coefficient* | 0.885 | 0.698 | 0.896 | 0.710 |
|  | *Cohen’s d* | 1.27 | 0.85 | 0.26 | 3.51 |
|  | *Paired t-test p-value* | 4E-74* | 3E-29* | 4E-11* | <E-100* |
| **FSL (L)** | | 1.320 ± 0.15 | 0.665 ± 0.08 | 0.492 ± 0.06 | 0.154 ± 0.03 |
| **FSL vs. FS** | FSL – FS *mean diff. (ml)* | -180.8 | -41.6 | 11.8 | NA |
|  | *Correlation Coefficient* | 0.872 | 0.786 | 0.960 | NA |
|  | *Cohen’s d* | -1.12 | -0.53 | 0.18 | NA |
|  | *Paired t-test p-value* | 7E-64* | 2E-54* | 5E-9* | NA |
| **FS (L)** | | 1.501 ± 0.17 | 0.706 ± 0.08 | 0.480 ± 0.07 | NA |
| **SPM vs. FS** | SPM – FS *mean diff. (ml)* | 16.6 | 23.4 | 27.0 | NA |
|  | *Correlation Coefficient* | 0.877 | 0.960 | 0.930 | NA |
|  | *Cohen’s d* | 0.1 | 0.31 | 0.44 | NA |
|  | *Paired t-test p-value* | 0.013* | 9E-9* | 1E-39* | NA |

Mean and standard deviation of the brain volumes estimated by SPM, FSL, and FS are presented. Cells corresponding to CSF_FS_ are filled as ‘NA’ since FS does not output total CSF volume. Inter-method differences and corresponding statistics are presented in shaded cells. Correlation coefficient is used to measure the association between the brain volumes estimated by two different methods. Cohen’s d is used to measure the effect size of the inter-method difference and paired t-test was used to check the statistical significance. Statistically significant differences are denoted by * for *p* < 0.05. Correlation coefficients show that methods agree on volume estimates but the Cohen’s d and paired t-test p-values indicate significant inter-method biases.
