# Supplementary material for: Inter-Method Discrepancies in Brain Volume Estimation May Drive Inconsistent Findings in Autism
Source: Front Neurosci. 2016 Sep 30;10:439. doi: 10.3389/fnins.2016.00439 (PMC5043189; doi:10.3389/fnins.2016.00439)
Supplement: Supplementary file 3 [file Table3.DOCX]

**Supplementary Table 3: ASD vs. TDC brain volume differences in NYU**

|  | **TIV** | | | **GM** | | | **WM** | | | **CSF** | | |
| --- | --- | --- | --- | --- | --- | --- | --- | --- | --- | --- | --- | --- |
|  | *diff*  *(ml)* | *diff*  *%* | *p-val* | *diff*  *(ml)* | *diff*  *%* | *p-val* | *diff*  *(ml)* | *diff*  *%* | *p-val* | *diff*  *(ml)* | *diff*  *%* | *p-val* |
|  | *Raw Brian Volumes* | | | | | | | | | | | |
| **SPM** | 5.17 | 0.34 | 0.84 | 9.3 | 1.28 | 0.42 | -4.4 | -0.87 | 0.63 | 0.29 | 0.11 | 0.96 |
| **FSL** | -15.4 | -1.16 | 0.52 | 1.7 | 0.25 | 0.90 | -12.6 | -2.54 | 0.20 | -7.1 | -4.52 | 0.14 |
| **FS** | -44.1 | -2.9 | 0.10 | 5.4 | 0.77 | 0.66 | -10.8 | -2.22 | 0.31 | NA | NA | NA |
|  | *Adjusted for age and sex* | | | | | | | | | | | |
| **SPM** | -0.81 | -0.05 | 0.97 | 2.4 | 0.33 | 0.81 | -4.89 | -0.96 | 0.52 | 1.69 | 0.60 | 0.76 |
| **FSL** | -22.5 | -1.70 | 0.28 | -15.0 | -2.26 | 0.16 | -14.4 | -2.89 | 0.10 | -2.7 | -1.73 | 0.36 |
| **FS** | -51.9 | -3.42 | 0.03* | -10.1 | -1.44 | 0.34 | -10.3 | -2.12 | 0.24 | NA | NA | NA |
|  | *Adjusted for age, sex, and FIQ* | | | | | | | | | | | |
| **SPM** | 10.9 | 0.72 | 0.61 | 8.1 | 1.12 | 0.41 | -0.8 | -0.15 | 0.92 | 3.6 | 1.28 | 0.52 |
| **FSL** | -8.9 | -0.67 | 0.66 | -9.5 | -0.53 | 0.37 | -9.3 | -1.43 | 0.28 | -1.3 | -1.87 | 0.66 |
| **FS** | -37.0 | -2.44 | 0.12 | -3.6 | -0.52 | 0.72 | -5.2 | -1.08 | 0.55 | NA | NA | NA |

Mean (ASD – TDC) difference (*diff*) in brain volume estimates according to SPM, FSL, and FS.

Percentage ASD vs. TDC group difference was calculated as $100$*($\bar{ASD}- \bar{TDC})/\bar{TDC}$, where $\bar{TDC}$ is the group mean of the TDC subjects. Cells corresponding to CSF_FS_ are filled as ‘NA’ since FS does not output total CSF volume. Statistically significant differences are denoted by * for p<0.05. ASD vs. TDC differences are dependent upon the method used and only in SPM TIV, GM and CSF volumes in ASD were significantly larger than TDC.
