# Supplementary material for: Inter-Method Discrepancies in Brain Volume Estimation May Drive Inconsistent Findings in Autism
Source: Front Neurosci. 2016 Sep 30;10:439. doi: 10.3389/fnins.2016.00439 (PMC5043189; doi:10.3389/fnins.2016.00439)
Supplement: Supplementary file 4 [file Table4.DOCX]

**Supplementary Table 4: Differential bias for diagnostic group (ASD) in NYU**

|  | TIV | | | GM | | | WM | | | CSF | | |
| --- | --- | --- | --- | --- | --- | --- | --- | --- | --- | --- | --- | --- |
|  | ***bias*** *(ml)* | ***bias***  ***%*** | ***beta***  ***p-val*** | ***bias*** *(ml)* | ***bias***  ***%*** | ***beta***  ***p-val*** | ***bias*** *(ml)* | ***bias***  ***%*** | ***beta***  ***p-val*** | ***bias***  *(ml)* | ***bias***  ***%*** | ***beta***  ***p-val*** |
| SPM vs. FSL^#^ | 12 | 1.64 | 0.13 | 17 | 2.6 | 0.09 | 10 | 1.9 | 0.09 | 4.4 | 2.90 | 0.36 |
| FSL vs. FS^#^ | 21 | 1.96 | 0.09 | -5 | 0.70 | 0.23 | -4 | 0.85 | 0.30 | NA | NA | NA |
| SPM vs. FS^#^ | 51 | 3.4 | 0.001* | 12.5 | 1.8 | 0.09 | 5.4 | 1.1 | 0.13 | NA | NA | NA |

**^#^** reference method

***bias (ml):*** brain volume (in ml) by which a method overestimates in ASD subjects than in TDCs.

***% bias:*** the percentage of brain volume by which a method overestimates in ASD subjects than in TDCs.
